# Supplementary material for: Are carbon emissions trading and green financial instruments synergistic? -Comprehensive quantitative research based on content analysis
Source: PLoS One. 2024 Mar 7;19(3):e0298601. doi: 10.1371/journal.pone.0298601 (PMC10919601; doi:10.1371/journal.pone.0298601)
Supplement: S1 Data — (ZIP) [file pone.0298601.s001.zip › Data/地级市Z_F工作报告.zip!/╦─┤¿/π≥╓▌╩╨/└┤╫╘╣1⁄2╓┌║┼í╛┬φ┐╦╩2╛▌═°í┐_2016.docx]

各位代表：

现在，我代表市人民政府，向大会报告工作，请予审查，并请市政协委员提出意见。

2015年工作回顾

2015年，是充满挑战、成果丰硕的一年。面对复杂严峻的经济形势，在省委、省政府和市委的正确领导下，在市人大、市政协的监督支持下，我们紧紧围绕“产业转型发展年”的工作主题，主动适应新常态，主动作为稳增长，经济实现平稳较快发展，社会保持和谐稳定。全市完成地区生产总值1353.4亿元、比2014年（下同）增长11%，经济总量在全省排名提升2位，跃居第6位；规模以上工业增加值增长14%，全社会固定资产投资1463.7亿元、增长23.9%，社会消费品零售总额559.7亿元、增长13.9%，地方一般公共预算收入128.3亿元、同口径增长10.3%，城镇居民人均可支配收入26656元、增长8.6%，农村居民人均可支配收入11359元、增长10.3%。在此，我向大会报告，市七届人大五次会议确定的主要目标任务较好完成，向全市人民承诺的民生实事全部兑现！

一、着力稳增长，经济总量实现跨越升位

精准施策稳增长。积极应对经济下行压力，突出重点领域精准发力。在工业经济、企业融资、投资促进、要素价格管理、房地产建筑业发展等方面，出台103条政策措施，促进了全市经济平稳较快发展，经济总量在全省排名提升2位，跃居第6位。

投资拉动稳增长。实施340个重点项目，完成投资1099.1亿元、增长33%。激发民间投资活力，完成民间投资980.6亿元、增长12.1%。叙古高速公路叙永至古蔺县城段主体工程完工，叙宜高速公路加快建设。叙大铁路叙永段投入运营，川南城际铁路内自泸段、隆黄铁路叙永至镇雄段开工建设。

扩大消费稳增长。积极组织企业参加市场拓展“三大活动”。全市实现商品零售476.5亿元、增长14.4%。新培育限额以上批发零售企业63户。大力促进住房消费，房地产开发健康有序，完成商品房销售面积403.4万平方米、增长32%。大力提升旅游休闲消费，努力扩大教育文化体育消费，积极促进健康养老家政消费。

开放合作稳增长。成功举办“2015中国国际酒业博览会”、第三届中国（泸州）西南商品博览会、“2015中国医药企业家年会”等重大投资促进活动。与韩国平昌郡、德国雷姆沙伊德市签署友好合作协议。着力招大引强，引进华为公司、南瑞集团、恒大集团、碧桂园集团等知名企业，到位市外国内资金929.5亿元、增长17.9%。启动实施“外向型经济三年攻坚行动”，进出口总额增长13.7%，到位外资增长11.7%。

要素保障稳增长。组建30亿元四川发展纯粮原酒股权投资基金，设立了18亿元规模的企业应急转贷资金。积极争取上级财政转移支付129亿元、国家专项建设基金29.7亿元、国家专项企业债券99亿元。成功获批全国第一支城市停车场项目收益债券。全市金融机构各项贷款余额1093亿元，较年初增加172.2亿元。争取各类用地指标2.3万亩。工业用电量39.7亿千瓦时，工业用气量12.9亿立方米，成品油供应53.2万吨。

二、着力调结构，产业转型升级取得重大突破

工业转型发展步伐加快。着力改造提升传统优势产业，白酒、化工、能源、机械四大产业增加值分别增长13%、9%、7.1%、18.8%。加快培育发展智能电网、大数据、高端装备制造、新能源新材料、现代医药等新兴产业。北辰电力产业项目建成投产，煤气化项目（一期）进入试车阶段，中海金象年产15万吨润滑油加氢项目试运行，步长生物制药及新药产业化基地、南瑞集团西部产业基地、华为四川大数据中心、东方雨虹新型防水材料基地、航天泸州工业园等项目开工建设。成功列入四川省绿色建材产业化示范基地、“互联网+制造”区域试点城市。

现代服务业优化升级。华润万象汇开业运营，西南商贸中心（二期）建材市场试营业。泸州临港产业物流园创建为国家电子商务示范基地，泸州电子商务中心（一期）建成运营。全市电子商务交易额135亿元、增长43%。泸州港保税物流中心（B型）封关试运行，泸州进口商品保税直销中心（一期）建成投运。成功创建为全国综合运输服务示范城市。泸州港完成集装箱吞吐量44.5万标箱、增长39%。泸州机场通航航线达16条，实现旅客吞吐量84.5万人次、增长18.9%。引进中航工业集团，组建幸福泸州航空有限公司。天津银行、成都银行在我市设立分支机构进展顺利。玉蟾温泉国际度假区（一期）、欢乐派海滩公园等旅游项目建成运营。花田酒地被评定为国家4A级旅游景区。纳溪区成功创建全国休闲农业与乡村旅游示范县（区）。全市实现旅游总收入253亿元、增长37.6%。

现代农业提质增效。扎实推进国家现代农业示范区建设，新建“千亿增收示范工程”万亩示范区11个，建成国家级、省级农业标准化示范项目38个，粮食总产量202.8万吨。新培育市级农业产业化龙头企业29家。新增农民专业合作社省级示范社17个。引进广东温氏集团100万头生猪一体化产业项目。山东寿光蔬菜产业集团、中国海升果汁控股有限公司入驻泸州。合江锁口水库、叙永倒流河水库主体工程完工，泸县三星桥水库开工建设。

“三大园区”竞相发展。泸州高新区创建为国家高新区，主营业务收入增长30.5%。泸州长开区创建为省级经济开发区，主营业务收入增长20%。泸州白酒产业园区实现扩区扩能和品质提升，主营业务收入增长20.1%。

三、着力抓统筹，新型城镇化建设迈出坚实步伐

新型城镇化综合试点有序推进。探索建立农业转移人口市民化成本分担机制、城镇化投融资机制，积极创新行政管理模式。泸县列入全国农村土地制度改革试点县，叙永、古蔺列入全国县城深化基础设施投融资体制改革试点县。纳溪大渡口镇、合江九支镇被列为全国建制镇示范试点镇。12个乡撤乡设镇，2个镇改设街道办事处。完成城镇基础设施投资72.7亿元、增长14.6%。建筑业实现总产值575.3亿元、增长10.9%。全市城镇化率达46.1%。

城市功能不断完善。积极推进“两江新城”建设，大力改造提升老城区。忠山隧道、蜀泸大道四期竣工投用，西南医疗康健中心（一期）、城北公交枢纽站主体工程完工，医教园区、城西城市综合体、沱江六桥及连接线等项目加快建设，长江六桥及连接线工程、沱江新城南滨公园和北滨公园开工建设。列入国家智慧城市试点市、四川智慧城市示范市，建成全国第一个全光网大城市。中心城区建成区面积达120.1平方公里、人口达120.4万人。

城市管理水平持续提升。加快建设智慧交通、智慧城管，城市精细化管理水平逐步提高。城乡环境综合治理有效开展，积极推进“三大专项治理行动”，城管综合执法体制机制不断完善。扎实开展全国文明城市创建工作。顺利通过国家卫生城市第六次复审。

四、着力促改革，体制机制创新纵深推进

行政体制改革不断深化。全面取消市本级非行政许可审批项目，清理公布市、县行政权力清单和责任清单，推进行政权力依法规范公开运行。深化政府机构改革，完善农业监督管理体制。实施商事制度改革，积极开展“先照后证”改革试点，“三证合一、一照一码” 改革有序推进，新增各类市场主体3.2万户、增长14.7%。在全国率先开展不动产统一登记示范建设工作，颁发了全国首批不动产权证书。

经济体制改革深入实施。深化财政体制改革，开展合理划分市区事权范围试点。依法将政府债务分类纳入预算，实行限额管理。泸州农村商业银行组建工作进展顺利。国资国企改革加快推进，泸州老窖集团公司与股份公司的管理体制进一步理顺；泸天化集团改革脱困取得新成效，股份公司实现扭亏为盈。深化投融资体制改革，完成采购阶段PPP项目4个，总投资95亿元。

创新能力得到加强。深入推进“大众创业、万众创新”，整合创新创业引导资金2.4亿元、人才引进基金1亿元，新建“众创空间”40个，建成省级科技企业孵化器5家。新增国家高新技术企业13家，实现高新技术产值156亿元、增长42%。实施科技成果转化项目53项，新增产值47.1亿元。我市成功获批国家知识产权试点城市，江阳区列入国家可持续发展示范区。

五、着力惠民生，民生事业取得新成效

民生实事全面完成。全年民生支出213亿元，占一般公共预算支出的70.3%。十项民生工程和20件民生大事全部兑现。城镇新增就业4.4万人，城镇登记失业率3.53%。城乡居民医疗保险合并实施。各项社会保险参保人数达765万人次。开工建设保障性住房1960套，实施危旧房棚户区改造16072套。启动实施“渡改桥”三年攻坚行动，开工建设渡改桥7座。实施地质灾害避险搬迁1209户。

社会事业协调发展。教育领域综合改革不断深化，素质教育深入实施，义务教育均衡发展有序推进，教育质量稳步提升。医药卫生体制改革全面推进，公共卫生保障能力持续增强。计生服务管理水平不断提高。市人民医院新院加快建设。全民预防保健试点工作得到中央领导的关注和批示。文体广电事业协调发展。市民中心主体工程完工，体育生态园加快建设。食品药品安全监管得到加强。

脱贫攻坚扎实推进。坚持区域扶贫与精准扶贫“双轮驱动”，大力推进“七大扶贫攻坚行动”，落实“五个一批”要求，投入专项扶贫资金2.1亿元，脱贫解困8.6万人。新改建县乡道397.8公里、通村公路713.5公里。解决了33.2万农村人口饮水安全问题、5.3万户群众用电难问题。

生态环保持续加强。强化节能减排，大气质量不断改善，可吸入颗粒物（PM10）比2013年（考核基准年）下降9.1%。全国水生态文明城市建设试点工作通过中期评估。泸州市垃圾焚烧发电厂试运行。新建乡镇污水处理厂24个。治理水土流失面积138平方公里。管护国有林55.1万亩，补偿集体公益林318.6万亩，巩固退耕还林61.5万亩，森林覆盖率达50%。

社会保持和谐稳定。认真落实安全生产责任，扎实开展安全生产大检查大整治，安全生产形势持续稳定，成为全省未发生较大以上安全生产事故的4个市州之一。深入开展“打黑除恶暨社会治安专项整治行动”，严厉打击各种违法犯罪活动，社会治安平稳可控。稳步推进信访制度改革，信访秩序进一步好转。

国防动员、人民防空、民兵预备役建设不断加强。妇女儿童、青少年、慈善、残疾人、老龄事业持续发展，对口援藏、民族宗教、台湾事务、外事、侨务、统计、消防、保密、档案、气象、地方志等工作取得了新成绩。

各位代表，过去一年，我们扎实开展“三严三实”专题教育，集中整治政府系统“不作为、慢作为、乱作为”，切实加强政府自身建设，政风行风明显改进，行政效能持续提升，联系群众更加紧密。法治政府建设深入推进，依法行政水平进一步提高。“六五”普法圆满完成。自觉接受市人大、市政协的监督，严格执行人大决议、决定。办理人大代表建议117件、政协委员提案313件，办结率100%。加强党风廉政建设，强化行政监察和审计监督，严厉查处政府系统违纪违法案件251起，始终保持惩治腐败高压态势。

各位代表，2015年全市经济社会发展取得的成就，是省委、省政府和市委正确领导的结果，是市人大、市政协监督支持的结果，是全市上下真抓实干的结果。在此，我代表市人民政府，向全市人民致以崇高的敬意！向人大代表、政协委员，向各民主党派、工商联、各人民团体，向驻泸部队官兵和社会各界人士，表示衷心的感谢！

在肯定成绩的同时，我们也清醒地看到，发展中还存在一些困难和问题。一是经济结构性矛盾还比较突出，产业层次低、创新能力不强等问题依然存在。二是改革开放任重道远，体制机制活力不强，开放型经济规模不大。三是保障和改善民生任务繁重，脱贫攻坚任务艰巨，安全生产和信访维稳形势仍然严峻。四是工作作风有待进一步转变，少数政府机关和公务人员存在“怠政”“懒政”等现象。对这些困难和问题，我们将采取有力措施，切实加以解决。

“十三五”时期总体目标任务

“十二五”时期，是泸州经济社会爬坡上行、提档升级的五年，开创了发展新局面，为在更高起点上加快发展奠定了坚实基础。五年间，全市经济总量在全省排名上升3位，全社会固定资产投资由第11位跃升至第2位，地方一般公共预算收入由第6位跃升至第2位。

未来五年，是我市“决胜全面小康、建成区域中心”的关键时期。按照市委的建议，市政府组织编制了《泸州市国民经济和社会发展第十三个五年规划纲要（草案）》。“十三五”时期，我们要深入学习贯彻习近平总书记系列重要讲话精神，紧紧围绕“四个全面”战略布局，牢固树立创新、协调、绿色、开放、共享的发展理念，坚定“着力六个突破、实现两个率先”总体目标，主动适应新常态，统筹推进经济、政治、文化、社会、生态文明建设，建成全国重要区域性综合交通枢纽、长江上游产业转型升级示范区、川滇黔渝结合部区域中心城市、生态文明美丽泸州。

“十三五”时期的主要目标任务：地区生产总值年均增长9%以上，到2020年经济总量超过2000亿元。城乡居民收入到2020年达到或超过全国平均水平。一是建成全国重要区域性综合交通枢纽。推动轨道交通发展突破，加快高等级广覆盖公路网建设，建成长江上游川滇黔航运物流中心，打造区域航空运输中心和国际航空港。到2020年，铁路里程突破400公里，公路里程达到1.5万公里，集装箱吞吐量达到100万标箱，泸州机场旅客吞吐量达到175万人次。二是建成长江上游产业转型升级示范区。改造提升传统优势产业，积极培育新兴产业，大力发展现代服务业，加快推进农业现代化。到2020年，新兴产业增加值占规模以上工业增加值的比重达到20%，三次产业结构调整为10:57:33。三是建成川滇黔渝结合部区域中心城市。全面推进新型城镇化综合试点，完善城镇发展体系和城市服务功能，建设现代化大都市。到2020年，中心城区形成面积200平方公里、人口200万人的大城市骨架。四是建成生态文明美丽泸州。强化资源节约和环境保护，森林覆盖率达到51%，重要江河湖泊水功能区水质达标率80%以上，万元GDP综合能耗累计下降12%。五是共建共享幸福美好生活。构建合理有序的收入分配格局，完善社会保障体系，积极推进社会事业发展，提高社会文明程度，建成全国文明城市。六是全力推进脱贫攻坚。到2019年，全面消除绝对贫困。

各位代表，上述目标任务，综合考虑了未来发展的趋势和条件，是时代的要求、人民的期待。我们将科学把握发展规律，主动适应和引领新常态，锐意进取、开拓创新，奋力谱写泸州发展的辉煌篇章！

2016年工作安排

2016年，是“十三五”的开局之年。做好今年的政府工作，要牢牢把握“稳中求进、进中求好、好中求快”的工作基调，以“产业创新发展年”为工作主题，统筹稳增长、调结构、惠民生、防风险，全力推进“着力六个突破、实现两个率先”，不断开创泸州经济社会健康发展新局面。

2016年主要预期目标是：地区生产总值比2015年（下同）增长9%，规模以上工业增加值增长11%，全社会固定资产投资增长16%，社会消费品零售总额增长11.5%，地方一般公共预算收入同口径增长9%，城镇居民人均可支配收入增长9%，农村居民人均可支配收入增长10%。

围绕上述目标，重点做好以下工作：

一、专注发展定力，千方百计稳定经济增长

强化投资拉动。围绕国家重点投资领域，着力扩大投资规模，优化投资结构，提高投资效益。突出基础设施、产业发展、民生及社会事业、生态环保等领域，实施重点项目481个，完成投资1207亿元、增长16%。加快推进区域综合交通枢纽建设，确保叙古高速公路叙永至古蔺太平段、叙宜高速公路建成通车，加快推进川南城际铁路内自泸段、隆黄铁路叙永至镇雄段、泸州机场迁建工程等项目建设，加快渝昆铁路、叙威高速、泸永高速公路前期工作。

扩大消费需求。实施 “六大消费工程”，加快培育消费热点。大力发展白酒电商、跨境电商、农村电商、社区电商等新业态，电子商务交易额增长45%。实施“宽带乡村”工程，实现村村通宽带，促进信息消费。推进全国养老服务业综合改革试点，扩大养老消费需求。积极引导库存消化和市场开发，促进房地产市场健康发展。大力推广应用新能源汽车。

培育骨干税源。强化企业运行监测，完善要素保障联席会议制度，协调解决企业生产经营中的困难和问题。积极筹建产业发展基金。实施“小巨人”、“成长型”企业培育工程，完善“一企一策”帮扶机制。认真落实国家减税降费政策，帮助小微企业发展壮大。

二、加快产业转型升级，提升产业核心竞争力

大力发展现代制造业。优化提升白酒、化工、能源、机械四大产业，培育发展新能源新材料、高端装备制造、现代医药等新兴产业。以泸州老窖、郎酒为引领，优化白酒产品结构，实施原酒战略，打造“泸州酿”区域品牌。启动建设泸州老窖10万吨酿酒基地、中远物流4.8万立方米固态酿造智能化储酒基地。加快建设聚碳酸酯项目（一期）、年产1000吨乙基纤维素项目。确保合盛硅业扩能技改项目、观文煤矿投产运行。开采页岩气1亿立方米。确保智通机器人研发和生产基地、国机重工西南（泸州）产业园建成投产。加快建设航天泸州工业园、西南智能电网产业园、东方雨虹新型防水材料基地，规划建设四川临港装备产业园。推进化工医药融合发展。加快步长生物制药及新药产业化基地、维思达医疗器械及耗材生产、四川西蜀九锦现代中药生产基地等项目建设。

加快发展现代服务业。推进华为四川大数据中心、省信用联社异地灾备大数据中心项目建设。依托智慧城市建设，加快智慧产业发展。推进华润购物中心、西南商贸中心（二期）、泸州海吉星农产品国际商贸物流园等项目建设。加快国家电子商务示范基地建设，推动传统产业与电子商务融合发展。支持华西证券、兴泸水务、川南大市场有限公司等企业上市，支持议中管业、金鼎印务等企业在“新三板”或区域性场外市场挂牌。鼓励银行金融机构增加信贷投放。力争幸福泸州航空有限公司开航运营，泸州机场新开通2条航线，旅客吞吐量突破90万人次。大力发展文化产业，着力打造“一园四区”。加快建设中国酒镇·酒庄旅游区、玉蟾温泉国际度假区（二期）、太平古镇旅游综合开发等项目，推动旅游景区提档升级，确保旅游总收入突破300亿元。

积极发展现代农业。突出抓好 “八大特色农业产业”，粮食总产量稳定在200万吨以上，种植酿酒高粱70万亩、烤烟12.5万亩，新发展优质茶叶基地1万亩、竹林基地5万亩、核桃基地5万亩、油茶基地3万亩、精品水果基地2万亩。积极招引现代农业龙头企业，大力发展农产品精深加工和休闲农业，着力打造江阳区董允坝现代农业示范园、纳溪区都市生态农业示范园、合江县现代农业示范园。加快建设古蔺观文水库、叙永龙洞水库，积极推进烟区水源工程建设。加强山、水、田、林、路综合治理。严守耕地红线，耕地保有量达到38.4万公顷。建立健全农产品质量安全监管体系。认真做好第三次全国农业普查工作。

提升园区集聚功能。泸州高新区重点发展智能电网、大数据、高端装备制造、现代医药、新能源新材料五大产业，主营业务收入增长18%。泸州长开区积极争创国家级经济开发区，突出发展现代商贸物流、临港装备制造、新材料等主导产业，完成军民融合产业示范区规划，主营业务收入增长15%。泸州白酒产业园区积极争创省级经济开发区，主营业务收入增长15%。启动泸州空港产业园区规划建设。

三、加快新型城镇化建设，推进城乡一体发展

有序推进中心城区建设。完善骨干路网，加快长江六桥及连接线、沱江六桥及连接线、沱江二桥加宽改造工程、沱江新城南滨路和北滨路等项目建设。加快建设二环线。完成城西客运站主体工程，确保城北公交枢纽站、主城区第一批停车场建成投用。探索推进地下管廊、海绵城市建设。确保主城区污水管网建设及改造工程、南郊二水厂（一期）等项目竣工。加快西南医疗康健中心、医教园区、沱江新城南滨公园和北滨公园、长湿新城湿地公园（一期）、长江滨江路小关门至柏木溪段等项目建设。完成沙茜片区拆迁安置，加快上坝、二道溪、小市麻沙桥等老城和棚户区改造。实施城市建设项目95个，完成投资66.4亿元。

提升城乡治理水平。完善数字化城管系统，加强城市精细化管理，推动全国城市管理综合执法体制改革试点。大力开展主城区环境秩序专项治理行动，推进农村生活垃圾治理体系建设，力争创建4个省级“环境优美示范乡镇”、45个省级“环境优美示范村庄”。大力推进全国文明城市创建，巩固提升国家卫生城市、国家园林城市创建成果。

抓好新型城镇化综合试点。坚持以人的城镇化为核心，稳步推进农业转移人口市民化，探索建立行政管理创新和行政成本降低的新型管理模式，统筹实施投融资机制改革、城乡规划管理体制机制改革。推进“宜居县城行动”，加快县城和重点集镇基础设施、公共服务能力建设，提高城镇化水平和质量。加快纳溪大渡口镇、合江九支镇等7个试点镇建设。大力实施幸福美丽新村“五大行动”。

四、全面深化改革开放，持续增强发展动能

深化重点领域改革。加快商事制度改革，大力推进注册资本登记制度、“三证合一、一照一码”、“先照后证”等改革。积极推进供给侧改革，不断激发市场活力。深化国有企业改革，稳步推进泸天化集团改革脱困。推进国有资产证券化。深化投融资体制改革，加强融资平台建设，发挥PPP项目投资基金作用，鼓励社会资本参与公共项目建设运营。推进地方法人金融机构改革，力争泸州农村商业银行挂牌。加快农村土地承包经营权确权登记，积极稳妥实施农村集体资产股份制改革、农户土地承包经营权入股及规范流转、农村土地流转收益保证贷款等试点。

提升开放合作水平。深化国际合作，积极参与全省“一带一路”战略“251三年行动计划”。深化沿江经济合作，围绕产业互接、通道共建、信息共享，加强与重庆、上海等沿江沿海城市合作。深化与毗邻地区合作，积极打造川渝合作示范区，推动赤水河流域合作发展。发挥泸州港保税物流中心（B型）、国家进境粮食指定口岸等优势，促进外向型经济发展。办好“2016中国国际酒业博览会”和第四届中国（泸州）西南商品博览会。坚持“三为主”招商模式，力争到位市外国内资金突破千亿元大关。

大力实施创新驱动。深入开展“大众创业、万众创新”，优化完善扶持政策，多渠道、多形式推动创新创业。加快推进知识产权试点城市建设，建成“川南科技成果与专利技术实体交易市场”。实施标准化战略，开展商标战略进园区活动。建设科技创新服务平台，积极打造“智慧园区”和“众创空间”，建成一批孵化中心、企业服务中心和大学生创业园。充分发挥泸州高新区孵化器作用，启动泸州长开区、白酒产业园区孵化器建设。大力引进教育、医疗卫生等专业人才，进一步加大政策支持，激励科技人才创新创业。

五、全力推进扶贫攻坚，加快脱贫致富步伐

深入实施区域扶贫。以古蔺、叙永、合江三个贫困县为主战场，实施连片扶贫开发，推进“七大扶贫攻坚行动”，集中力量破解基础设施、产业发展等难题。统筹解决 “插花式”贫困问题，确保江阳、龙马潭、纳溪、泸县4个区县全面脱贫。力争全年减少贫困人口10万人以上。

扎实抓好精准扶贫。按照“六个精准”、“五个一批”要求，严格实施“一村一计、一户一策”，确保项目到村、扶贫到户。扎实推进教育扶贫、医疗扶贫、文化扶贫、旅游扶贫、电商扶贫。新增农村转移劳动力5.9万人。切实抓好易地扶贫搬迁，实现搬迁安置1.6万人。落实救济救助、低保等政策，兜底保障特殊贫困群体基本生活。

健全扶贫工作机制。坚持“扶贫与防返贫” 相结合，进一步完善扶贫资金使用、督查考核、驻村帮扶等机制。注重“输血与造血” 相结合，充分发挥扶贫资金撬动作用、合作组织纽带作用、贫困群众主体作用，确保扶贫工作取得实效。持续推进“定点扶贫”、“扶贫结对网络行”、“百企联百村”活动，进一步凝聚脱贫攻坚力量。

六、加强和创新社会治理，切实增进民生福祉

深入实施民生工程。持续加大民生投入，确保公共财政用于民生支出的比重保持在70%左右。全面落实就业促进政策，城镇新增就业3.6万人，城镇登记失业率控制在4.0%以内。进一步完善社会保障体系，扩大社会保险覆盖面，提高社会保障水平。大力推进“渡改桥”三年攻坚行动，开工建设渡改桥37座，建成10座。完成重大地灾工程治理4处、中小型地灾排危除险15处、避险搬迁1361户。强化食品药品监管，确保泸州食品药品检测中心建成投用，积极创建国家食品安全示范城市。

全面发展社会事业。积极推进教育综合改革试验区建设，大力改善办学条件，加快教育信息化进程，努力促进教育均衡与教育公平。繁荣文化艺术创作生产，实施舞台艺术精品工程、振兴川剧百戏工程。加快推进公共文化服务体系建设，实施“高清泸州·智慧广电”工程。办好泸州市第七届运动会。深化医药卫生体制改革，夯实基层医疗基础。加强重大疾病防控。全面实施“两孩”政策。积极创建健康城市。力争完成市人民医院新院主体工程。积极推进全民预防保健服务体系建设。认真做好新一届全国双拥模范城创建迎检工作。完成第十届村（居）委会换届工作，稳妥推进农村社区建设试点。加强国防动员和国防教育，强化国防后备力量建设。切实做好民族宗教、老龄、台湾事务、外事、侨务、保密、档案、气象、人防、地方志等工作。

切实维护安全稳定。深入贯彻执行《安全生产法》，全面落实“五级五覆盖”、“五落实五到位”，夯实安全生产基层基础，强化安全隐患动态排查治理，加强应急救援体系、防灾减灾体系建设，持续开展“打非治违”专项行动，确保人民生命财产安全。积极推进“阳光信访、责任信访、法治信访”建设，提高信访工作法治化、规范化和信息化水平。加强社会治安综合治理，开展专项整治行动，依法严厉打击违法犯罪活动，加强社区矫正对象、吸毒人员等特殊群体管控。积极支持工会、共青团、妇联、残联等人民团体参与社会治理。

着力建设“生态文明美丽泸州”。巩固退耕还林成果，加强天保工程（二期）、新一轮退耕还林、石漠化治理、国家储备林基地建设，加强水土流失治理。加快推进全国水生态文明城市创建，落实最严格的水资源管理“三条红线”制度。强化重点行业污染整治，抓好大气、水和土壤污染防治，深入开展“两江四河”综合治理，全面完成节能减排目标任务。

七、加强政府自身建设，增强政府执行力和公信力

加快法治政府建设。严格执行市人大及其常委会决议、决定，自觉接受市人大、市政协监督，认真办理人大代表建议和政协委员提案。广泛听取民主党派、工商联、无党派人士、人民团体的意见和建议，自觉接受社会监督。依法行使权力、履行职责，严格执行重大行政决策程序规定。加强政府立法工作，建立政府立法体制机制。完善行政执法体制，积极推进城市管理、城乡规划建设、农业、水务等综合执法工作。

加快效能政府建设。进一步简政放权，全面推行“三张清单”制度。切实加强执行力建设，强化对重大决策、重点工作落实情况的跟踪督查，确保政令畅通、执行有力。建设政务大数据互联互通平台，加强电子政务与网站建设，强化行政效能电子监察。加强政务公开监管，继续推进行政权力依法规范公开运行。

加快廉洁政府建设。全面落实从严治政各项规定，坚持“三严三实”标准，持续整治“四风”，巩固提升“为民、务实、清廉”的政府形象。严格落实党风廉政建设责任制，抓好省委巡视组反馈问题整改落实，健全完善土地出让、项目建设、招投标、国有资产管理等机制，切实加强行政监察和审计监督。加大政府系统违纪案件查处力度，坚决整治损害群众利益的不正之风，打造风清气正的政务环境。

各位代表，回顾“十二五”，辉煌成就令人鼓舞；展望“十三五”，宏伟蓝图催人奋进。泸州发展已站在新的历史起点上，让我们紧密地团结在以习近平同志为总书记的党中央周围，在市委的坚强领导下，开拓创新、奋勇拼搏，为实现泸州“决胜全面小康、建成区域中心”而努力奋斗！
